# Exosomes derived from pericardial adipose tissues attenuate cardiac remodeling following myocardial infarction by Adipsin-regulated iron homeostasis

**DOI:** 10.3389/fcvm.2022.1003282

**Published:** 2022-09-12

**Authors:** Wanrong Man, Xinglong Song, Zhenyu Xiong, Jing Gu, Jie Lin, Xiaoming Gu, Duan Yu, Congye Li, Mengyuan Jiang, Xuebin Zhang, Zhi Yang, Yang Cao, Yan Zhang, Xiaofei Shu, Dexi Wu, Haichang Wang, Gang Ji, Dongdong Sun

**Affiliations:** ^1^Department of Cardiology, Xijing Hospital, Fourth Military Medical University, Xi’an, China; ^2^School of Basic Medicine, Fourth Military Medical University, Xi’an, China; ^3^Department of Physiology and Pathophysiology, Fourth Military Medical University, Xi’an, China; ^4^Department of Radiation Oncology, Xijing Hospital, Fourth Military Medical University, Xi’an, China; ^5^Heart Hospital, Xi’an International Medical Center, Xi’an, China; ^6^Department of Gastrointestinal Surgery, Xijing Hospital of Digestive Diseases, Fourth Military Medical University, Xi’an, China

**Keywords:** myocardial infarction (MI), exosomes, pericardial adipose tissue, ferroptosis, Adipsin

## Abstract

As a vital adipokine, Adipsin is closely associated with cardiovascular risks. Nevertheless, its role in the onset and development of cardiovascular diseases remains elusive. This study was designed to examine the effect of Adipsin on survival, cardiac dysfunction and adverse remodeling in the face of myocardial infarction (MI) injury. *In vitro* experiments were conducted to evaluate the effects of Adipsin on cardiomyocyte function in the face of hypoxic challenge and the mechanisms involved. Our results showed that Adipsin dramatically altered expression of proteins associated with iron metabolism and ferroptosis. *In vivo* results demonstrated that Adipsin upregulated levels of Ferritin Heavy Chain (FTH) while downregulating that of Transferrin Receptor (TFRC) in peri-infarct regions 1 month following MI. Adipsin also relieved post-MI-associated lipid oxidative stress as evidenced by decreased expression of COX2 and increased GPX4 level. Co-immunoprecipitation and immunofluorescence imaging prove a direct interaction between Adipsin and IRP2. As expected, cardioprotection provided by Adipsin depends on the key molecule of IRP2. These findings revealed that Adipsin could be efficiently delivered to the heart by exosomes derived from pericardial adipose tissues. In addition, Adipsin interacted with IRP2 to protect cardiomyocytes against ferroptosis and maintain iron homeostasis. Therefore, Adipsin-overexpressed exosomes derived from pericardial adipose tissues may be a promising therapeutic strategy to prevent adverse cardiac remodeling following ischemic heart injury.

## Introduction

Myocardial infarction (MI) refers to an acute and devastating myocardial anomaly characterized by occlusion of coronary perfusion and cardiomyocyte cell death. Acute MI remains one of the leading causes of mortality worldwide, claiming millions of lives each year, and poses enormous challenge to public health (1). Despite the advance in coronary artery revascularization to open occluded vessels, many MI patients still develop adverse cardiac remodeling and heart failure. Cardiomyocyte death in the infract region is a crucial pathogenic factor for the gradual development of deterioration of heart function following MI (2, 3). Although necrosis, apoptosis, necroptosis and pyroptosis have been indicated in the onset and development of adverse cardiac remodeling and cardiomyocyte cell death (4, 5), limited strategies are readily available to restore cardiomyocyte survival and improve prognosis in MI patients. When acute MI occurs, there are still abundant cardiomyocytes with metabolic activity residing in the peri-infarct region (6). Thus, effective measures to preserve peri-infarct cardiomyocytes are pertinent to combat unfavorable cardiac remodeling following MI insult.

Adiposity disorders and ischemic heart disease are intertwined with one another. Adiposity is known to worsen clinical outcome of ischemia-reperfusion (IR) injury, although molecular mechanism(s) remains to be established. Adipokines are secreted proteins from adipose tissues with an essential role in ischemic heart disease (7, 8). For example, deranged circulatory adipokines including leptin, tumor necrosis factor-α, transforming growth factor-β, and adiponectin are often noted in obese individuals, contributing to a time-dependent post-MI cardiac injury (7). In this context, adipokines capable of preserving cardiomyocyte survival may be of particular importance in the realm of post-MI heart injury.

Adipsin, an adipokine also known as complement factor D (CFD), was first described in 1987 with a key role in the rate-limiting step of alternative complement pathway (9). Multiple preclinical studies have shown a role for Adipsin in pathological conditions such as ischemia reperfusion and sepsis (10, 11). In addition, Adipsin is shown to preserve beta cell function and energy homeostasis in diabetes mellitus. Adipsin serves as a novel prognostic biomarker in patients with coronary artery diseases (12). Nonetheless, the role of Adipsin in the post-MI cardiac remodeling injury remains elusive.

Exosomes are endosome-derived nanovesicles with diameters ranging from 40 nm to 160 nm. Exosomes carry encapsulated payloads such bioactive proteins, non-coding RNAs, and lipids, and are recognized as essential mediators of intercellular communication (13). The exosomal membrane prevents breakdown of these bioactive compounds, ensuring their long-term stability. Because of their surface protein profile, exosomes possess a high target selectivity for receptor cells. Exosomes are a crucial mediator for intracellular and inter-organ communication (14, 15). Exosomes are also recognized as a reasonable drug delivery vehicle, with potential applications in disease treatment. Exosomes, with unique biological properties, play important roles in adipokines-based intercellular and inter-organ crosstalk.

To this end, this study was designed to explore the role of Adipsin in MI and post-MI cardiac remodeling and injury, if any, as well as the underlying mechanisms involved.

## Materials and methods

### The determination of Adipsin

A clinical observational survey was conducted in Xijing hospital to determine the changes of serum Adipsin levels in patients with acute MI. This survey was approved by the Institutional Review Board of Xijing Hospital and was in accordance with the principles outlined in the Declaration of Helsinki. All participants were given written informed consent (ClinicalTrials.gov Identifier: NCT04570527). From October 2020 to April 2021, a total of 14 patients with acute MI were involved in this study. Serum was collected at the time of admission and 1 month later. Samples were analyzed in triplicate applying a human Adipsin ELISA kit (Abcam, ab243686). In terms of the mice, Adipsin was determined using WB or ELISA kits (R&D Systems, DY5430-05) in tissues and serum.

### Animals

All procedures were performed in adherence to the Fourth Military Medical University’s *Guidelines on the Care and Use of Laboratory Animals* and were approved by the Ethics Committee on Animal Care. Adipose tissue-specific Adipsin transgenic mice (Adipsin-Tg) and adipose tissue-specific deletion of Adipsin mice (Adipsin-KO) were generated on C57BL/6J background provided by Shanghai Model Organisms Center (Shanghai, China). Male littermates (6–8 weeks, 20–25 g) were utilized in all animal experiments.

### Animal study protocol

Myocardial infarction surgery was performed under anesthesia (2% isoflurane inhalation). Left anterior descending (LAD) branch of coronary artery 2–3 mm from its origin was permanently ligated by a knot around with silk suture (16). Sham mice were subjected to similar surgical procedures except for ligation of coronary artery. MI surgery was performed 2 weeks after adeno-associated virus injection. Exosome treatments were performed *via* tail vein after MI surgery.

One month after MI surgery, observational and histological analyses were made. Mice were euthanized using 30% VD/min of 100% carbon dioxide in adherence to *The 2020 AVMA Guidelines on Euthanasia.*

### Isolation and characterization of exosomes

Pericardial, epididymal and subcutaneous adipose tissues were harvested from the Adipsin-Tg, Adipsin-KO or control mice (NTg and WT). Tissues were sliced into small pieces of less than 0.1 cm^3^ and were cultured for 24 h in serum-free DMEM culture media (17, 18). Exosomes were isolated from supernatant of culture media. Debris and cells in the supernatant were removed by centrifugation at 1,000 *g* for 5 min and then at 10,000 *g* for 10 min. After filtering with a 0.22 μm filer, exosome isolation was carried out using the ultracentrifuge technique or an Exoquick kit (19). To characterize exosomes, size distribution analysis was performed on diluted exosomes (1 mg/mL for protein) by NanoSight (Malvern, United Kingdom). Transmission electron microscopy (TEM) (JEM-2000EX TEM, Japan) was used to evaluate the morphological characteristics of exosomes.

### Exosome tracking *in vivo*

Exosomes (approximately 1 μg/μl protein level) were treated for 30 min with 1 mM DiR (Invitrogen D12731) in a volume ratio of 500:1. Free dyes were cleared by centrifugation. DiR-labeled exosomes (100 μg at protein level) were injected into mice with indicated treatments *via* tail veins. Exosome localization in the body and individual organs was detected by Pearl Trilogy Small Animal Imaging System (LI-COR, United States) 12 h after injection.

### Echocardiography

M-mode Echocardiography was conducted using an echocardiography system with a 15-MHz linear transducer (Visual Sonics Vevo 3100, Toronto, ON, Canada). Left ventricular end-diastolic diameter (LVEDD) and left ventricular end-systolic diameter (LVESD) were measured. Each measurement was made with M-mode from three consecutive beats. Left ventricular ejection fraction (LVEF) and left ventricular fraction shortening (LVFS) were calculated by computer algorithms.

### Masson trichrome stain

Interstitial fibrosis was evaluated using Masson trichrome staining. Hearts were fixed overnight in 4% paraformaldehyde (pH 7.4), embedded in paraffin, and were serially sectioned at 5μm thickness. Trichrome Masson Stain Kit (Sigma, HT15) was employed for staining. To measure collagen deposits, sections were stained with methyl blue. Fibrosis was measured by an Image J software (National Institutes of Health, NIH).

### Immunohistochemistry and Perls’ Prussian Blue staining

For tissue fixation, wax blocks were cut into 5 μm thick sections. Slides were deparaffinized, and subjected to antigen retrieval in hot citric acid buffer. After cooling, slides were permeabilized with 0.15% Triton X-100 for 15 min and were blocked with 1% BSA in PBS for 2 h, prior to incubation overnight with primary antibody (4°C). Slides were then incubated for 1 h with anti-rabbit secondary antibody (1:500 dilution, Proteintech, SA00003-2) at room temperature. The primary antibodies used included: anti-Adipsin rabbit polyclonal antibody (ab213682, 1/200). Non-heme iron staining of heart tissues was measured using a Perls’ Prussian Blue stain kit (Solarbio, G1422) (20, 21).

### Cardiac tissue non-heme iron and malondialdehyde content

The chromogen method was used to analyze non-heme iron (22). The NHI acid (1M HCl and 10% trichloroacetic acid in high-purity water) was used to digested cardiac tissue for 48 h at 65°C. Samples were cooled in water at room temperature for 5 min, vortex mixed, and then centrifuged at 10,000 g for 10 min. The supernatant and chromogen solution (0.2% thioglycolic acid and 0.02% disodium-4,7-diphenyl-1,10-phenanthroline desulphonated in 50% saturated NaAc solution) were mixed in a ratio of 1:1. After 20 min at room temperature, samples were read at 562 nm. Standard curves were prepared by iron standards (0, 2, 4, 6, 8 and 10 μg/ml of the iron standard solution). Standard curves were linear with slopes ranging from 0.12 to 0.15 AU/(Ag Fe/ml). Results were given in micrograms of iron per gram of wet tissues.

Malondialdehyde (MDA) is a widely used marker of oxidative lipid injury. In this study we used the commercial kit (Beyotime, S0131), which was based on measurement of thiobarbituric acid (TBA).

### Isolation of neonatal mouse ventricular cardiomyocytes

Primary cultures of neonatal mouse ventricular cardiomyocytes (NMVMs) were prepared from neonatal C57BL/6J mice. Hearts were removed immediately following euthanasia, and were subjected to enzyme digestion reagent (1.0 mg/mL collagenase type II, Gibco, #17101015). Cells were cultured in DMEM (Hyclone, SH30022.01) containing 10% fetal bovine serum (FBS), 0.1 mM 5-Bromo-2’-deoxyuridine (BrdU, Sigma, B5002), and 1% penicillin/streptomycin and maintained at 37°C in 5% CO_2_. On the following day, medium was replaced with DMEM containing 10% FBS without BrdU.

### Cell culture and *in vitro* treatment

Cardiomyocytes were incubated at 37°C in a humidified atmosphere containing 5% CO_2_ and 95% air. *In vitro*, hypoxic environment was simulated with modular hypoxia chamber. Oxygen level was less than 2% in the hypoxia chamber. Cardiomyocytes were pretreated with recombinant Adipsin (10 μg/ml) or vehicle (saline).

### RNA sequencing and data analysis

Whole-genome gene expression analysis was performed using neonatal ventricular cardiomyocytes exposed to Adipsin- and saline in hypoxia atmosphere (*n* = 3 per group). The RNA was extracted using Trizol (Invitrogen, #15596026), and cDNA samples were sequenced by a sequencing system (HiSeq3000; Illumina). The gene information was downloaded from the National Center for Biotechnology Information database. Heat map analyses were used to express different genes. Kyoto Encyclopedia of Genes and Genomes (KEGG) ontology enrichment analyses were also performed. Differences with a *p*-value < 0.05 were considered statistically significant.

### Immunoprecipitation and mass spectrometric analysis

Immunoprecipitation was performed on cell protein samples using a Pierce Classic Magnetic IP/Co-IP Kit (Thermo Fisher Scientific, #88828) according to the manufacturer’s protocol. Adipsin protein complexes were immunoprecipitated with anti-Adipsin antibody-conjugated beads by SDS-PAGE gel and were then stained by Silver Staining Kit (Beyotime, P0017S). The silver-stained bands were determined using LC-MS/MS. The spectra were assessed by the National Center for Biotechnology Data protein database with Mascot and Sequest. Local alignments were determined using Multiple Sequence Comparison by Log-Expectation (MUSCLE).

### Co-immunoprecipitation

Cardiomyocytes were treated with recombinant Adipsin (10 μg/mL) or vehicle for 6 h under hypoxia. Cells were washed once with cold PBS, and lysed with cold lysis buffer (Beyotime, #S0131) supplemented with a protease inhibitor cocktail (Thermo Fisher Scientific, 78438). Cell lysates were incubated with an anti-Adipsin antibody (Abcam, ab213177, 3 μg) and were incubated at 4°C for overnight. The protein A beads were rinsed with a lysis buffer. Proteins were eluted from the beads and were resolved by IgG elusion buffer (Thermo Fisher Scientific, 1856202). Samples containing reducing agent (dithiothreitol) were heated and separated using electrophoresis. Following transfer to polyvinylidene fluoride membranes, proteins were immunoblotted with an anti-Irp2 rabbit polyclonal antibody (23829-1-AP, 1/800). Co-immunoprecipitation (Co-IP) was performed using a Pierce Classic Magnetic IP/Co-IP Kit (Thermo Fisher Scientific) according to the manufacturer’s protocol.

### Downregulation of target genes by adeno-associated virus 9 (AAV9)

First, small interfering RNAs were designed and synthesized by Tsingke Biotechnology Company (Beijing, China). Downregulation of target gene was performed by transfection of cardiomyocytes with Irp2-specific small interfering RNA *in vitro*. Next, the siRNA sequences that had been confirmed were transformed into shRNA sequences via BLOCK-iT RNAi Designer^1^. For animal experiments, adeno-associated virus 9 carrying Irp2-shRNA (AAV9-Irp2-shRNA) and control vector were constructed by Hanbio Technology (Shanghai, China). Downregulation of Irp2 was performed by intramyocardial injection of AAV9-Irp2-shRNA *in vivo* (2.0E + 10 genome copies, 20 μl per mouse).

### Immunofluorescence

For analysis of immunofluorescence, cardiomyocytes were incubated with the anti-Adipsin (Abcam, ab121054, 1/1,000), anti-Irp2 (23829-1-AP, 1/800) antibodies followed by staining with Alexa Fluor 488-conjugated anti-mouse IgG (1:200, Ab150113), Alexa Fluor 647-conjugated anti-rabbit IgG (1:200, Ab150075) antibodies. Positive cells were quantified using an Image-Pro Plus software (Media Cybernetics, United States) and detected by confocal microscopy (Nikon, Japan).

### Quantitative PCR

Total RNA was extracted from cells or tissues using Trizol (Invitrogen, 15596026). RNA level was measured using a spectrophotometry. The cDNA was prepared from 1μg RNA using the PrimeScript RT reagent Kit (Takara, RR047A) in accordance with the manufacturer’s instruction. Quantitative PCR was performed using a StepOnePlus™ Real-Time PCR System (Thermo Fisher Scientific 4376600) and TB Green Premix DimerEraser (Takara, RR081) in accordance with the manufacturer’s instructions. Primers were purchased from Takara Biomedical Technology (Supplementary Table S1). Fold difference in the gene expression was calculated using the 2^–ΔΔ*Ct*^ method and Gapdh mRNA served as housekeeping targets. All experiments were performed in triplicate, and specificity was monitored using the melting curve.

### Western blot analysis

Protein samples were collected from tissues or cells with RIPA Lysis Buffer (50 mM Tris pH 7.4, 150 mM NaCl, 1% Triton X-100, 1% sodium deoxycholate, 0.1% SDS). Protein was loaded onto SDS-PAGE gel electrophoresis system (PowerPac, Bio-Rad, 164-5052). PVDF membranes were first used for transfer prior to membrane blocking with 5–8% skimmed milk for 1 h. They were then incubated with primary antibodies for 8–12 h (4°C), and were finally incubated with secondary HRP-conjugated antibodies (anti-mouse, Proteintech #SA00001-1, 1/10,000; anti-rabbit, Proteintech #SA00001-2, 1/10,000). WB densitometry was quantified using the Image Lab system. Antibodies used included anti-Adipsin rabbit monoclonal antibody (Abcam, ab213177, 1/1,000), anti-transferrin rabbit monoclonal antibody (Abcam, ab109503, 1/8,000), HRP anti-beta actin mouse monoclonal antibody (Abcam, ab20272, 1/5,000), anti-TFRC rabbit polyclonal antibody (Proteintech, 10084-2-AP, 1/2,000), anti-ferritin heavy chain rabbit monoclonal antibody (Abcam, ab183781, 1/1,000), anti-COX2 rabbit polyclonal antibody (ABclonal, A1253, 1/3,000), anti-GPX4 rabbit monoclonal antibody (ABclonal, A11243, 1/1,000), anti-GAPDH rabbit polyclonal antibody (Proteintech, 10494-1-AP, 1/8,000).

### Statistical analysis

Statistical analyses were performed using the SPSS 20.0 software package (IBM Corp., Armonk, NY, United States). Information visualization was completed using the R program (Version 3.3.6). Statistical analyses of variations between teams were made by the Student’s *t*-test, one-way ANOVA with a Fisher’s *post hoc* comparison test or two-way ANOVA with multiple *post hoc* comparisons. Differences with a *p*-value < 0.05 were considered statistically significant.

## Results

### Myocardial infarction increases Adipsin expression during the acute phase

**FIGURE 1:**
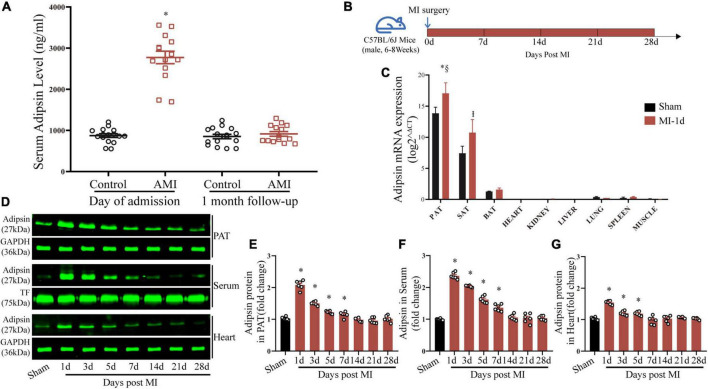
Acute myocardial infarction evokes Adipsin production and secretion. **(A)** Serum Adipsin levels at admission and 1 month after discharge in MI patients. *n* = 16 and 14, *p < 0.05 vs. Control group. **(B)** Schematic illustration of animal experimental procedures analyzing Adipsin production and secretion. **(C)** Adipsin mRNA expression determined in pericardial adipose tissue (PAT), subcutaneous adipose tissue (SAT), brown adipose tissue (BAT) and other organs using RT-qPCR. *n* = 6, *p < 0.05 vs. Sham group (PAT), I*p* < 0.05 vs. Sham group (SAT), §*p* < 0.05 vs. MI group (SAT). **(D–G)** Adipsin protein levels of pericardial adipose tissue (PAT), serum and heart tissues determined using Western blots. *n* = 6 (sham) and 42 (MI). *p < 0.05 vs. Sham group. For **(A,C,E,F,G)**, statistical analysis was performed using one-way ANOVA.

To decipher the potential role of Adipsin in the realm of MI, serum Adipsin levels were determined in patients with acute MI. Upon admission, Adipsin levels in the serum were significantly higher in acute MI patients than the control group (Figure 1A and Supplementary Table S1). At the 1-month follow-up, the differences in serum Adipsin levels between groups vanished (Figure 1A).

We then established an experimental MI mouse model and sampled them for 4 weeks in order to investigate the changes of Adipsin production and secretion *in vivo* (Figure 1B).

Adipsin mRNA expression was examined using real-time quantitative PCR (RT-qPCR) in tissues or organs, including pericardial adipose tissue (PAT), subcutaneous adipose tissue (SAT), brown adipose tissue (BAT), heart, kidney, liver, lung, spleen, and muscle, to determine the source of Adipsin production. Our findings indicated that the predominant source of serum Adipsin production is adipose tissues. Additionally, PAT was more sensitive to MI, with the greatest degree of Adipsin upregulation following MI (Figure 1C).

Adipsin protein levels in PAT were significantly elevated, as indicated by Western blot analysis (Figures 1D,E). In our MI model, a similar trend was seen for serum and cardiac tissues following MI injury (Figures 1D,F,G). MI-evoked changes in Adipsin expression were also observed using immunohistochemistry technique in adipose tissues from mice subjected to MI (Supplementary Figure S1).

It is worth mentioning that Adipsin protein levels in cardiac tissues depend on serum levels (Figures 1D,F,G), while Adipsin mRNA expression in cardiac tissues is fairly low (Figure 1C). This implies that Adipsin found in cardiac tissues is most likely to originate from adipose tissues prior to delivery *via* systemic circulation.

### Accumulation of exosomes derived from pericardial adipose tissues (Pericardial-AT Exo)

**FIGURE 2:**
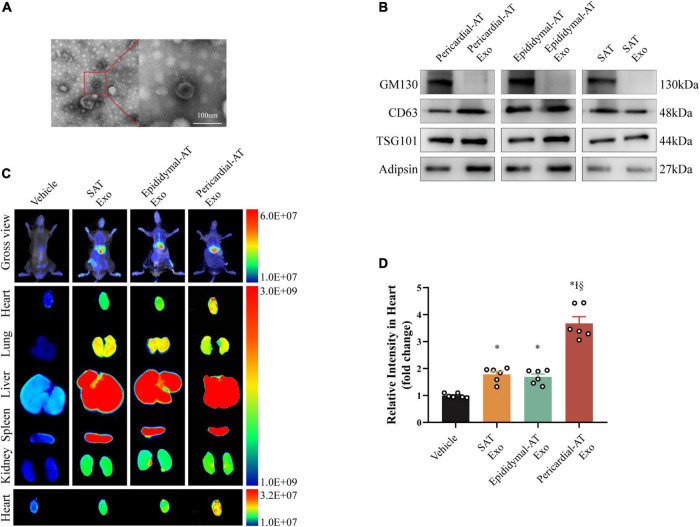
Exosomes from pericardial adipose tissue circulate into heart efficiently. **(A)** Electron micrographs of exosomes. Scale bar = 100 nm. **(B)** Inclusive and exclusive markers of exosomes in pericardial adipose tissue (Pericardial-AT), epididymal adipose tissue (Epididymal-AT) and subcutaneous adipose tissue (SAT), including GM130, CD63, TSG101, and Adipsin. **(C)** Imaging of DiR-labeled exosomes from different tissues. Exosomes from pericardial (Pericardial-AT), epididymal (Epididymal-AT) and subcutaneous adipose tissue (SAT) labeled with DiR was injected *via* tail vein. About 12 h after injection, fluorescence imaging was performed *in vivo* and *ex vivo*. **(D)** Quantification of relative fluorescence intensity of infiltrated exosomes in hearts. Each group *n* = 6. *p < 0.05 vs. Vehicle group, I*p* < 0.05 vs. SAT Exosomes group, §*p* < 0.05 vs. Epididymal-AT Exosomes group. Statistical analysis was performed by one-way ANOVA.

Given that pericardial fat releases a substantial quantity of adipokines and is linked to cardiac function (23, 24), we focused on exosomes derived from pericardial adipose tissues (Pericardial-AT Exo) to investigate the effect of Adipsin on the heart. Under electron microscopy, exosomes isolated and purified from pericardial adipose tissues had a characteristic exosome shape *in vitro* (Figure 2A and Supplementary Figure S2). In addition, WB analysis of isolated exosomes showed the exosome positive markers (CD63 and TSG101) were highly expressed, while the exclusive protein GM130 was unidentified. Adipsin protein was detected in these exosomes (Figure 2B).

Exosomes were labeled with DiR and were injected *via* tail veins to investigate the enrichment potential of Pericardial-AT Exo in diverse organs. They were heavily concentrated in the heart 12 h after intravenous injection (Figure 2C). Notably, exosomes derived from other adipose tissues did not exhibit similar cardiac enrichment capacity at the same injected dosage (Figures 2C,D). Furthermore, WB analysis of cardiac tissues revealed that exosomes derived from pericardial adipose tissues transported Adipsin to myocardial tissues (Supplementary Figure S2).

### Pericardial-AT Exo of Adipsin overexpression mice alleviate myocardial infarction-induced cardiac injury

**FIGURE 3:**
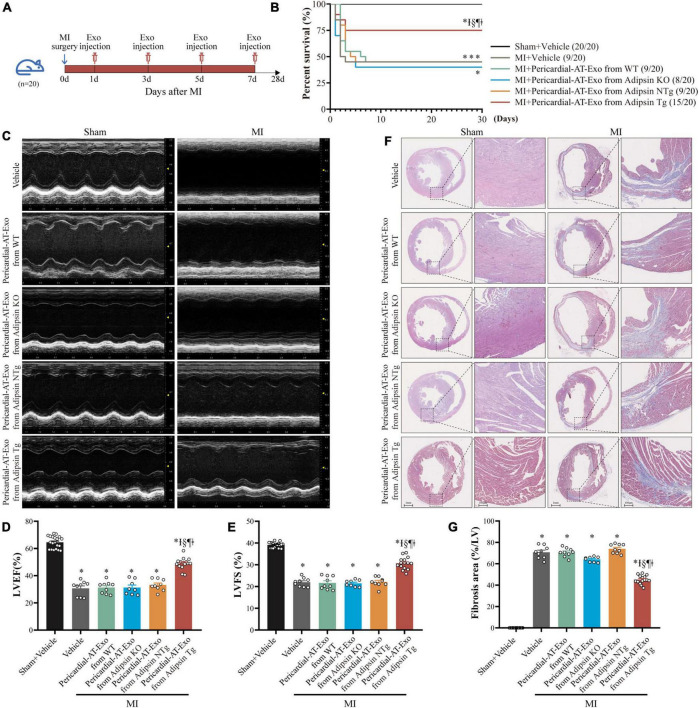
Pericardial-AT Exo of Adipsin overexpression mice alleviate MI-induced cardiac injury. **(A)** Schematic illustration of experimental procedure. MI mice were injected exosomes isolated from pericardial adipose tissue of donor mice (WT, Adipsin-KO, Adipsin-NTg, and Adipsin-Tg) on the first, third, fifth and seventh day *via* tail veins after MI surgery. Echocardiography and hearts collection were done 28 days after MI surgery. **(B)** Survival curves. Each group *n* = 20. *p < 0.05 vs. Sham + Vehicle group, I*p* < 0.05 vs. MI + Vehicle group, § *p* < 0.05 vs. MI + Pericardial-AT-Exosomes from WT mice group, ¶*p* < 0.05 vs. MI + Pericardial-AT Exosomes from Adipsin-KO mice group, ‡*p* < 0.05 vs. MI + Pericardial-AT Exosomes from Adipsin-NTg mice group. **(C)** Representative echocardiographic images 28 days after MI surgery. **(D,E)** Left ventricular ejection fraction (LVEF) and fractional shortening (LVFS) of each group (*n* = 8–20). *p < 0.05 vs. Sham + Vehicle group, I*p* < 0.05 vs. MI + Vehicle group, §*p* < 0.05 vs. MI + Pericardial-AT-Exosomes from WT group, ¶*p* < 0.05 vs. MI + Pericardial-AT Exosomes from Adipsin-KO group, ‡*p* < 0.05 vs. MI + Pericardial-AT Exosomes from Adipsin-NTg group. **(F,G)** Representative images of Masson’s trichrome staining (transverse) and quantification of fibrotic area (*n* = 8–20). *p < 0.05 vs. Sham + Vehicle group, I*p* < 0.05 vs. MI + Vehicle group, §*p* < 0.05 vs. MI + Pericardial-AT-Exosomes from WT group, ¶*p* < 0.05 vs. MI + Pericardial-AT Exosomes from Adipsin-KO group, ‡*p* < 0.05 vs. MI + Pericardial-AT Exosomes from Adipsin-NTg group. Survival curves were analyzed using Gehan–Breslow–Wilcoxon test. Mean ± SEM, Data for **(D,E,G)** and one-way ANOVA was used for statistical analysis.

To evaluate the possible cardioprotective property of Adipsin in MI, Adipose tissue specific Adipsin overexpression and knockout mice were bred (Supplementary Figures S3, S4), and exosomes were isolated and purified from the pericardial adipose tissues of these mice. Next, in the acute stage of MI, model mice were given intravenous injection of exosomes (100 μg at protein level) generated from different lines (WT, Adipsin-KO, NTg, Adipsin-Tg), and the efficacy was judged 28 days later (Figure 3A). Our findings indicated that exosomes derived from Adipsin overexpression mice significantly increased the survival rate after MI surgery (Figure 3B and Supplementary Table S2).

It should be mentioned that exosomes derived from Adipsin-Tg mice preserved left ventricular ejection fraction (LVEF) and fractional shortening (LVFS) following MI challenge (Figures 3C–E). Simultaneously, myocardial pathological damage was also alleviated. Masson trichrome staining indicated that myocardial fibrotic area was overtly reduced following injection of exosomes (Adipsin-Tg) (Figures 3F,G).

### Iron metabolism and ferroptosis regulation facilitate Adipsin-elicited action

**FIGURE 4:**
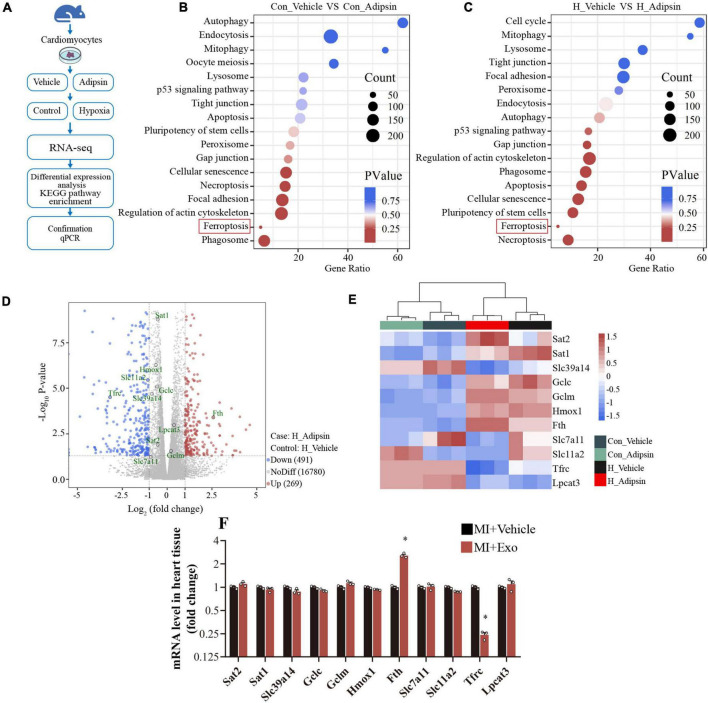
Adipsin regulates ferroptosis pathway *in vitro*. **(A)** Flowchart of the study design. Neonatal mouse ventricular cardiomyocytes were treated with recombinant Adipsin protein. Following that, cardiomyocytes were exposed to hypoxia for 6–8 h. Next, RNA sequencing was performed on the cell genome. **(B,C)** Pathway enrichment investigation. **(D)** Under hypoxia, the Volcano graph shows differentially expressed genes. **(E)** The Heatmap of differentially expressed genes about ferroptosis. **(F)** Using quantitative PCR, the expression profile of ferroptosis-related genes in cardiac tissue post MI was investigated after Adipsin-enriched pericardial-AT exosomes treatment. *n* = 3, *p < 0.05 vs. MI + Vehicle group. Data were mean ± SEM, and one-way ANOVA was used for statistical analysis.

To identify possible mechanisms for Adipsin-offered cardiomyocyte response to MI, neonatal mouse ventricular myocytes (NMVMs) were isolated and were treated with or without Adipsin under hypoxia or normoxia (Figure 4A). Then cellular transcriptome RNA was extracted and analyzed by RNA sequencing (Supplementary Table S3).

KEGG analysis noted that Adipsin protected cardiomyocytes against hypoxia injury (simulating MI injury) through regulation of ferroptosis (Figures 4B,C). Differential-expression analyses indicated that Adipsin evoked 269 upregulated genes and 491 downregulated genes in cardiomyocytes following hypoxia injury (Figure 4D). In addition, Adipsin treatment dramatically altered the expression of proteins associated with iron metabolism and ferroptosis, such as Tfrc, Fth, Slc11a2, Slc39a14, and Lpcat3 (Figure 4E). To verify the bioinformatic result, we went on to perform RT-qPCR analysis and found that Exo-Adipsin-Tg significantly enhanced levels of iron storage gene Fth *in vivo*. Adipsin reduced Tfrc expression, denoting its role in iron transportation (Figure 4F).

### Adipsin regulates iron homeostasis and reduces lipid oxidative stress in cardiac tissues after myocardial infarction injury

**FIGURE 5:**
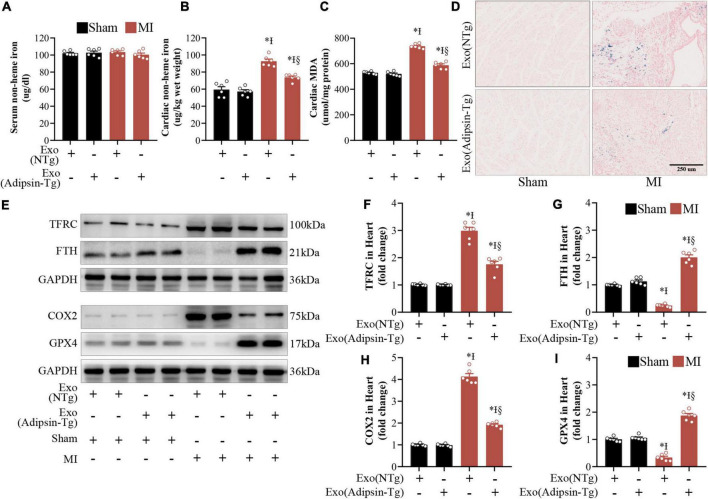
Adipsin-enriched pericardial-AT exosomes regulate iron homeostasis and alleviate lipid oxidative stress *in vivo*. **(A,B)** Non-heme iron levels in the serum and the peri-infarct region of the cardiac tissue were assessed in MI mouse after treatment with Adipsin-transgenic or non-transgenic exosomes. Each group *n* = 6, *p < 0.05 vs. Sham + Pericardial-AT exosomes from Adipsin-NTg mice, I*p* < 0.05 vs. Sham + Pericardial-AT exosomes from Adipsin-Tg mice, §*p* < 0.05 vs. MI + Pericardial-AT exosomes from Adipsin-NTg mice. Data were mean ± SEM, and one-way ANOVA was used for statistical analysis. **(C)** The levels of malondialdehyde (MDA) in cardiac tissue were measured in the peri-infarct area. Each group *n* = 6, *p < 0.05 vs. Sham + Pericardial-AT exosomes from Adipsin-NTg mice, I*p* < 0.05 vs. Sham + Pericardial-AT exosomes from Adipsin-Tg mice, §*p* < 0.05 vs. MI + Pericardial-AT exosomes from Adipsin-NTg mice. Data were mean ± SEM, and one-way ANOVA was used for statistical analysis. **(D)** Perls’ Prussian Blue-stained heart slices. **(E–I)** TFRC, FTH, COX2, and GPX4 proteins were measured by Western blots. Each group *n* = 6, *p < 0.05 vs. Sham + Pericardial-AT exosomes from Adipsin-NTg, I*p* < 0.05 vs. Sham + Pericardial-AT exosomes from Adipsin-Tg, §*p* < 0.05 vs. MI + Pericardial-AT exosomes from Adipsin-NTg. Data were mean ± SEM, and one-way ANOVA was used for statistical analysis.

To determine whether deranged iron homeostasis and ferroptosis play a role in cardiac responses elicited by Adipsin in MI injury, non-heme iron assay was utilized in serum and peri-infarct cardiac tissues from MI-challenged mice. Interestingly, Adipsin transgenic (Adipsin-Tg) exosomes decreased non-heme iron contents in cardiac peri-infarct regions, while exerting little effects on non-heme iron contents in serum (Figures 5A,B). These data indicated a specific regulatory effect of Adipsin on cardiac ischemic injury. Moreover, Adipsin-Tg exosomes exhibited lower iron contents in peri-infarct cardiac regions compared with NTg exosomes 1 month after MI injury as evidenced by the Perls’ Prussian Blue staining (Figure 5D). Consistent with our previous results, expression of FTH (iron storage associated protein) was upregulated, while the level of TFRC (cellular iron up-taking protein) was decreased in peri-infarct cardiac regions of mice 1 month after MI (Figures 5E–G). Malondialdehyde (MDA), an indicator of lipid oxidative stress, was evaluated in peri-infarct regions 1 month after MI. Adipsin-Tg exosomes decreased MDA levels as well (Figure 5C). Western Blots showed that Adipsin-Tg exosomes significantly decreased levels of COX2, indicating a beneficial role for Adipsin in post-MI-associated lipid oxidative stress (Figures 5H,I). These data revealed that exogenous administration of Adipsin mitigated iron over-loading and lipid oxidative stress in the peri-infarct region after MI injury.

### Iron homeostasis related protein (IRP2) may serve as a downstream regulator of Adipsin

**FIGURE 6:**
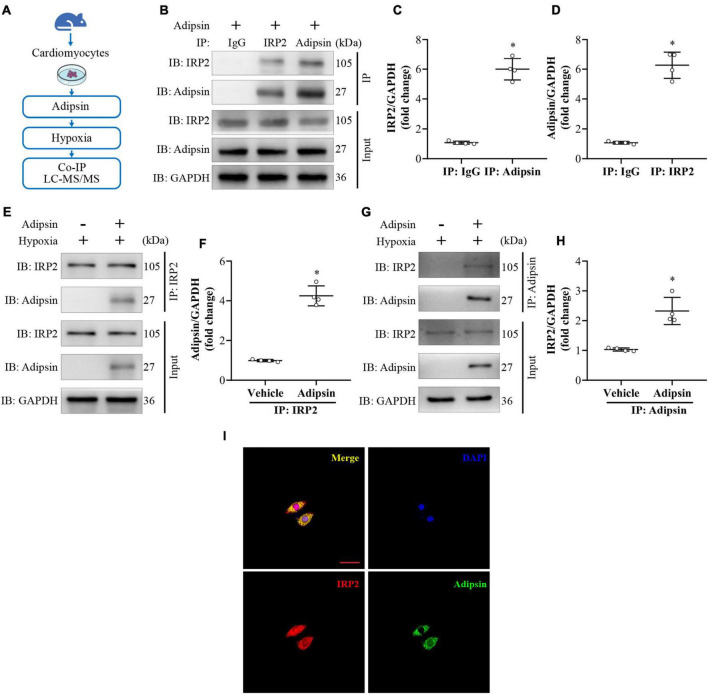
Adipsin interacts with IRP2 under hypoxia *in vitro*. **(A)** Flowchart of the study design. Neonatal mouse ventricular cardiomyocytes were treated with recombinant Adipsin protein. Following that, cardiomyocytes were exposed to hypoxia for 6–8 h. Next, Co-immunoprecipitation (Co-IP) and LC-MS/MS technologies were used to investigate cellular proteins. **(B–D)** Interaction between Adipsin and IRP2 was demonstrated using Co-IP. *p < 0.05 vs. IP: IgG. **(E–H)** Co-IP of Adipsin and IRP2 in cardiomyocyte in hypoxic environment. *p < 0.05 vs. Vehicle. **(I)** The Co-localization of Adipsin (green) and IRP2 (red) proteins in cardiomyocyte. DAPI, 4’,6-diamidino-2-phenylindole. Data were mean ± SEM, and one-way ANOVA was used for statistical analysis.

To elucidate downstream signaling mechanisms for Adipsin-regulated iron homeostasis and lipid oxidative stress, isolated NMVMs were subjected to Adipsin treatment under hypoxia (Figure 6A). Cell lysates were co-immunoprecipitated using anti-Adipsin antibody or IgG (control). Differentially expressed proteins were obtained using electrophoresis indicated by sliver staining. Liquid Chromatography Tandem-Mass Spectrometry (LC-MS/MS) analysis was used to analyze proteins which may directly interact with Adipsin (Supplementary Figure S5). Following LC-MS/MS analysis of the target band, the Adipsin-interacting protein iron homeostasis related protein (IRP2) was selected for further analysis (Supplementary Table S4).

To further verify the interaction between Adipsin and IRP2 in cardiomyocytes under hypoxia, we performed immunoprecipitation (IP) assays with different antibodies. As expected, an obvious interaction between Adipsin and IRP2 was detected (Figures 6B–D). Co-immunoprecipitation analysis further confirmed a direct interaction of Adipsin and IRP2 under hypoxia (Figures 6E–H). Immunofluorescence imaging also verified co-localization of Adipsin and IRP2 in cardiomyocytes (Figure 6I).

### IRP2 is essential to Adipsin-offered cardioprotection

IRP2 plays an essential role in iron homeostasis (25, 26). Under physiological conditions, the labile iron pool serves as the main source of mediating intracellular ROS and other important physiological function. As Adipsin interacts with IRP2 in our *in vitro* primary cardiomyocyte hypoxia model, the role of IRP2 in Adipsin-offered cardioprotection was determined.

To establish a causative relationship between IRP2 and Adipsin cardioprotection, IRP2 was knocked down using small interfering RNA (siRNA) in primary cardiomyocytes (Supplementary Figure S6). Adeno-associated virus 9 (AAV9) was used as a vector to construct AAV9-IRP2-shRNA that can knockdown expression of cardiac IRP2 (detailed steps were shown in Supplementary Figures S7–S9).

**FIGURE 7:**
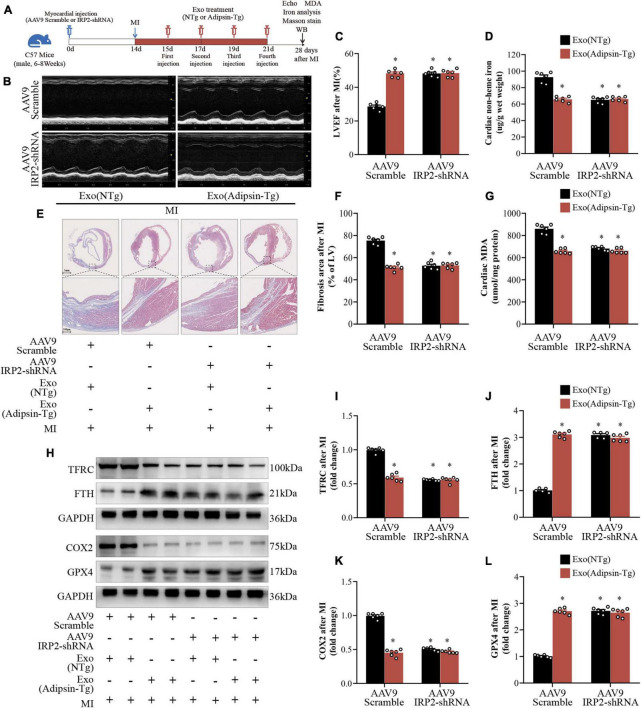
Irp2 is essential to Adipsin-offered cardioprotection. **(A)** Flowchart of the study design. Myocardial infarction surgery was performed 14 days after injection of AAV9 (Scramble or IRP2-shRNA) at the area of the myocardium. Following that, MI mice were injected four times with exosomes isolated from Adipsin-Tg or NTg mice’s pericardial adipose tissues. Cardiac function was assessed 28 days after MI surgery. Each group *n* = 6. **(B,C)** Representative echocardiographic images and left ventricular ejection fraction (LVEF) analysis. Each group *n* = 6. *p < 0.05 vs. AAV9-Scramble + Exo (NTg) group. **(D)** Non-heme iron levels were measured in the peri-infarct region of heart. Each group *n* = 6. *p < 0.05 vs. AAV9-Scramble + Exo (NTg) group. **(E,F)** Quantification of fibrotic area and representative images of Masson’s trichrome staining of the transverse planes post myocardial infarction. Each group *n* = 6. *p < 0.05 vs. AAV9-Scramble + Exo (NTg) group. **(G)** Malondialdehyde (MDA) levels in the peri-infarct region of heart. Each group *n* = 6. *p < 0.05 vs. AAV9-Scramble + Exo (NTg) group. **(H–L)** TFRC, FTH, COX2, and GPX4 proteins were measured by Western blots. Each group *n* = 6. *p < 0.05 vs. AAV9-Scramble + Exo (NTg) group. Data were mean ± SEM, and one-way ANOVA was used for statistical analysis.

The *in vivo* study design is shown in Figure 7A. Normal mice were injected with AAV9-IRP2-shRNA in cardiac tissues, and 2 weeks later, IRP2 was knocked down. Mice were subsequently given exosome treatment (Adipsin-Tg or NTg) four times after the MI surgery, and cardiac function and iron metabolism were evaluated 28 days later.

Compared with the control group (AAV9-Scramble + Exo-NTg), both AAV9- IRP2-shRNA and Exo-Adipsin-Tg treatment preserved heart function considerably. However, the combination of the two therapies did not enhance cardiac function any further (Figures 7B,C). Other pathological phenotypes of cardiac tissues, including non-heme iron, MDA contents, and myocardial fibrosis, revealed comparable tendencies (Figures 7D–G).

Western blots suggested that proteins associated with iron metabolism such as TFRC and FTH changed significantly in AAV9-IRP2-shRNA or Exo-Adipsin-Tg treated mice (Figure 7H–J). In addition, AAV9-IRP2-shRNA or Exo-Adipsin-Tg treated mice displayed significantly lower COX2 level and higher GPX4 level in response to MI challenge (Figure 7K,L).

These findings imply that both AAV9-IRP2-shRNA and Exo-Adipsin-Tg injection independently alleviated iron overload and lipid oxidation stress in cardiac tissues after MI. However, the two strategies cannot be combined to achieve a superposition effect. Based on these results, IRP2 may be a critical molecule for Adipsin-offered cardiac protection.

## Discussion

Although adipokines have been recognized as important regulators of cardiomyocyte survival and cardiac remodeling (27–29), the role of adipose tissues as an endocrine organ in the pathophysiology of MI may be underestimated (30). Certain adipokines including leptin, tumor necrosis factor-α, and resistin have been shown to evoke maladaptive cardiac remodeling following MI. To the contrary, adiponectin (APN) and C1q tumor necrosis factor-related proteins (CTRPs) offer protective action against myocardial ischemic injury and cardiac remodeling (31, 32). Due to the high prevalence of heart disease, it is pertinent to develop effective avenue for the cardiomyocyte-specific delivery of adipokines.

Exosomes, tiny membrane vesicles with diameters of 40—160 nm, emerge as a potential mediator for intercellular communication and drug delivery. Adipose tissue-derived exosomes have recently gained considerable attention. However, exosomes from different sources exhibit disparate affinity for organs and tissues (33–35). Our findings suggest that pericardial adipose-derived exosomes are more likely to be enriched in cardiac tissues.

As one of the first described adipokines, Adipsin was reported to play pivotal roles in gastrointestinal ischemia (GI) injury (10). Adipsin is considered to catalyze the rate-limiting step of alternative complement activation pathway in the activation of C3 and formation of the membrane attack complex (36, 37). However, recent evidence have suggested that Adipsin may regulate beta cell health independent of the classical Adipsin/C3a pathway. The effects of Adipsin on MI injury are still largely unknown. Our study presents the first piece of evidence for altered Adipsin levels in a subset of patients with MI. As confirmed by serum Adipsin levels in MI patients and mice with experimental MI, a significant rise in Adipsin levels is noted in acute MI. However, Adipsin levels gradually decline to the baseline level 28 days after MI. In this context, the degree of adiposity may not always be associated with increased cardiovascular risk. Patients with MI may be risk stratified based on their Adipsin levels, and those patients with the lowest Adipsin levels may need more aggressive anti-remodeling therapy to reduce their risk of developing adverse cardiac remodeling and heart failure. Alternatively, patients with high levels of Adipsin may be relatively protected from MI insult which warrants the clinical application of Adipsin administration.

Data from our work revealed a cardioprotective role for Adipsin in post-MI cardiac remodeling and functional derangement. Adipsin is an adipokine closely associated with the etiology of atherosclerosis. Ohtsuki and his team reported a role for Adipsin in predicting all-cause mortality and rehospitalization in patients with coronary artery disease (12). Importantly, these authors found that Adipsin is highly expressed in atherosclerotic plaque (12). However, a recent study have noted an opposite finding where double knock-out of Adipsin and LDL receptor in mice (Adipsin^–/–^:Ldlr^–/–^) triggered atherosclerosis (38). They have indicated that ablation of Adipsin may not be protective against atherosclerosis injury. Although results from these studies seem contradictory, the role of Adipsin in cardiovascular regulation appears to be essential and deserves further exploration.

Findings from our present study indicated that MI upregulated Adipsin levels in acute MI phase, and this phenomenon was observed in not only MI patients, but also mouse models of MI injury. In this context, adipose tissue-specific Adipsin transgenic mice (Adipsin-Tg) and adipose tissue-specific deletion of Adipsin mice (Adipsin-KO) were generated, and exosomes were isolated from pericardial adipose tissues of these mice and injected into MI mice. Interestingly, exosomes from Adipsin overexpression mice’s pericardial adipose tissues improved post-MI survival. Echocardiography and Masson trichrome staining indicated that Adipsin conferred protective properties against cardiac remolding post MI. These findings should shed some light on the role of Adipsin as a unique adipokine with diagnostic and therapeutic potential in cardiac remodeling after MI.

To determine possible cellular mechanisms responsible for the beneficial effect of Adipsin, RNA-seq analysis was performed and our data ranked iron metabolism and ferroptosis pathway atop of Adipsin-mediated responses in cardiomyocytes. In particular, exosome (Adipsin-Tg pericardial adipose tissue) treatment-maintained iron homeostasis and reduced lipid oxidative stress in the peri-infarct region *in vivo*.

Cardiac IR injury was documented to evoke cellular iron accumulation (39, 40). In many cardiopathic conditions, iron overload is deemed cardiotoxic as seen in our study of post-MI injury *in vivo*. This is in line with our observed changes in MDA, GPX4, iron storage and transporting proteins (Fth and Tfrc) in post-MI hearts from exosome (Adipsin-Tg) and vehicle treatment mice. Recent studies have also indicated a role for ferroptosis, a novel form of regulated cell death, in myocardial I/R injury. Ferroptosis contributes to cardiomyocyte loss in ischemic heart diseases. The fact that Adipsin mitigated MI-induced ferroptosis and iron metabolic derangement denotes an essential role for ferroptosis and iron metabolism in Adipsin-offered cardiac protection against MI.

**FIGURE 8:**
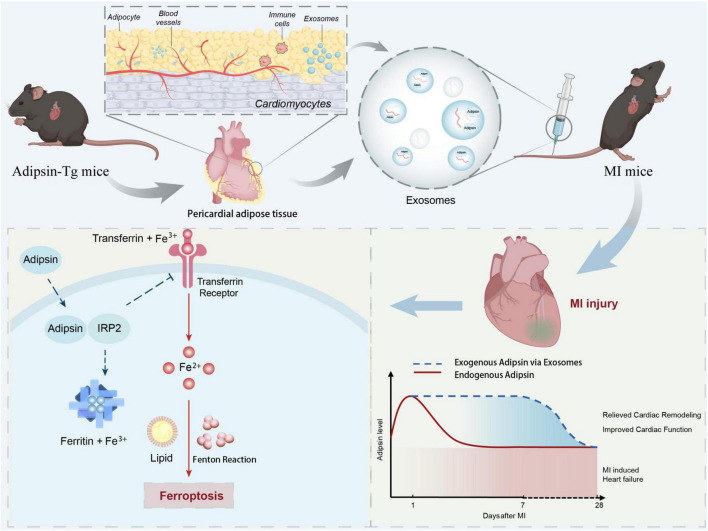
Schematic diagram. Illustration of time-course of Adipsin levels following MI challenge and the role of exogenous Adipsin-evoked cardioprotection. A reduction of Adipsin expression (red line) is visible during the initial stage of MI. Exogenous administration of Adipsin via exosomes (blue dashed line) triggers cardioprotection, relieves cardiac remodeling, and improves cardiac function. MI, myocardial infarction; IRP2, Iron regulatory protein 2; ROS, reactive oxygen species.

One intriguing finding from our present work is the involvement of iron-responsive element binding proteins (IRPs) as the key regulator of cellular iron pool and iron homeostasis (26, 41, 42). In our hands, Adipsin colocalizes and directly interacts with IRP2. Hypoxia exposure canonically mediates activation of the iron-starvation response *via* activation of IRP2, which would increase the stability of target mRNAs of iron uptake protein, such as transferrin receptor (TFRC), while inhibiting translation of other mRNAs encoding targets, such as iron storage protein ferritin (FTH). In this context, we have reasons to believe that the interaction between Adipsin and IRP2 may lead to inability of IRP2 and iron-responsive element binding, *en route to* decrease iron-starvation response and ferroptosis propensity. These data suggest that Adipsin acts, at least in part, via IRP2 to potentiate iron homeostasis modulation and ferroptosis inhibition (Figure 8).

In summary, data from our current work demonstrate that pericardial adipose tissue-derived exosomes can be efficiently delivered to myocardial tissues. Adipsin interacts with IRP2 to protect cardiomyocytes against ferroptosis and maintain iron homeostasis, and thus injection of Adipsin-overexpressed pericardial adipose-derived exosomes may be considered as a promising therapeutic avenue in the protection of hearts against MI injury.

## Data availability statement

The raw data presented in the study are deposited in the Sequence Read Archive (SRA) repository, accession number: SUB11928853.

## Ethics statement

The studies involving human participants were reviewed and approved by Institutional Review Board of Xijing Hospital. The patients/participants provided their written informed consent to participate in this study. This animal study was reviewed and approved by Fourth Military Medical University’s Guidelines on the Care and Use of Laboratory Animals.

## Author contributions

DS defined the topic of this project and revised the manuscript carefully. WM, XSo, ZX, and DY carried out the laboratory experiments and wrote the manuscript. JL, YZ, YC, XZ, XSh, ZY, and DW analyzed the data. GJ translated the literature and polished the manuscript. CL and XG were responsible for the animal model establishment. All authors carried out this work, read and approved the final manuscript.

## Footnotes

1
http://rnaidesigner.thermofisher.com/rnaiexpress/
