# Supplementary material for: Exosomes derived from pericardial adipose tissues attenuate cardiac remodeling following myocardial infarction by Adipsin-regulated iron homeostasis
Source: Front Cardiovasc Med. 2022 Sep 12;9:1003282. doi: 10.3389/fcvm.2022.1003282 (PMC9510661; doi:10.3389/fcvm.2022.1003282)

Figure 1

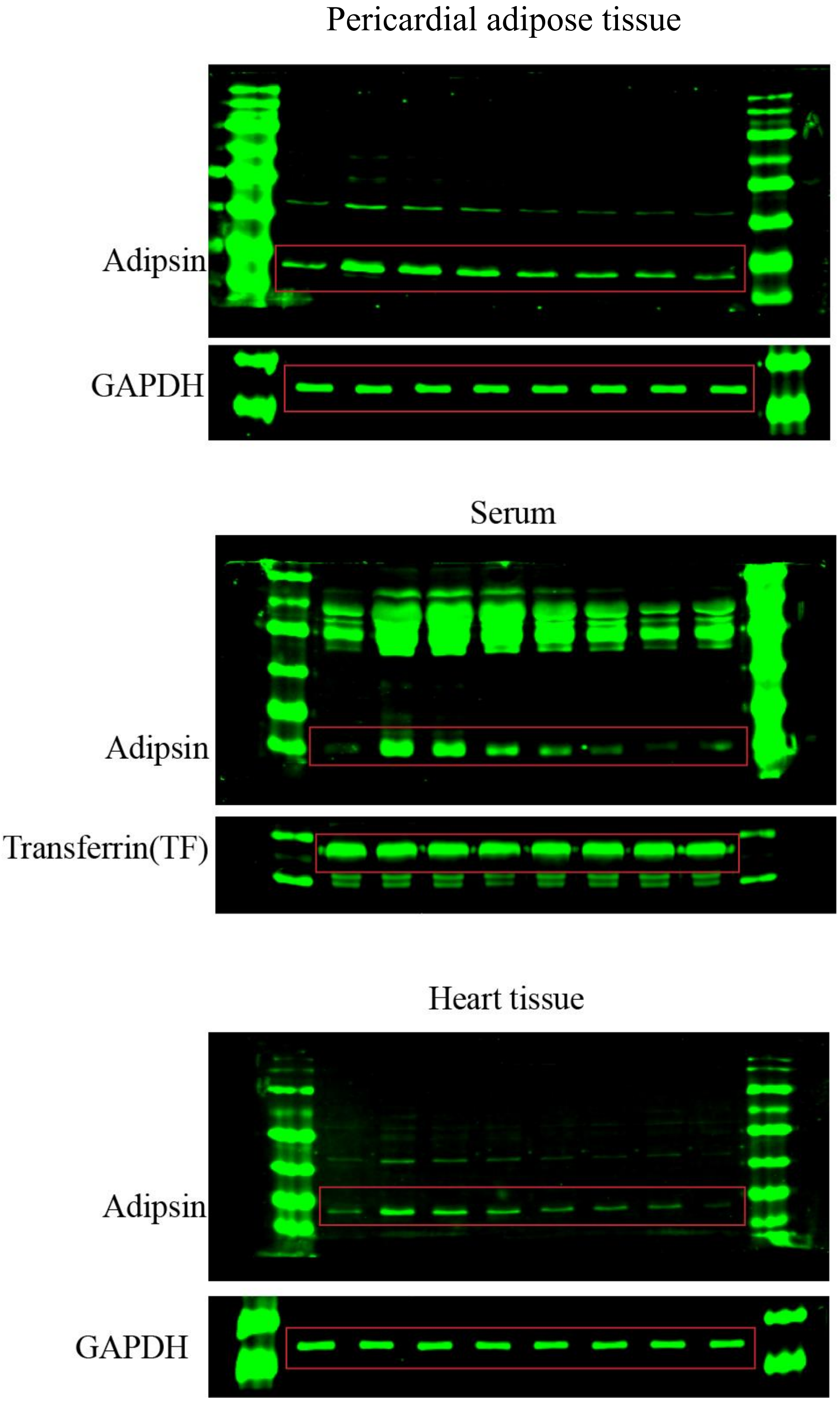
Pericardial adipose tissue
Adipsin
GAPDH
Serum
Adipsin
Transferrin(TF)
Heart tissue
Adipsin
GAPDH

Figure 2

GM130

CD63

TSG101

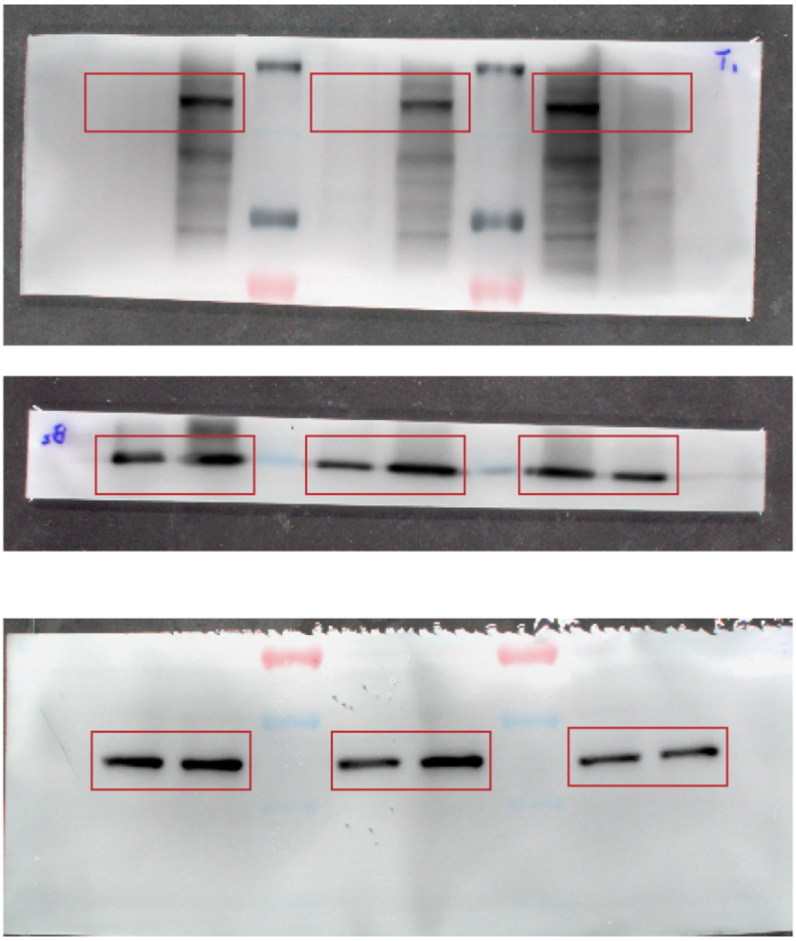

Figure 5

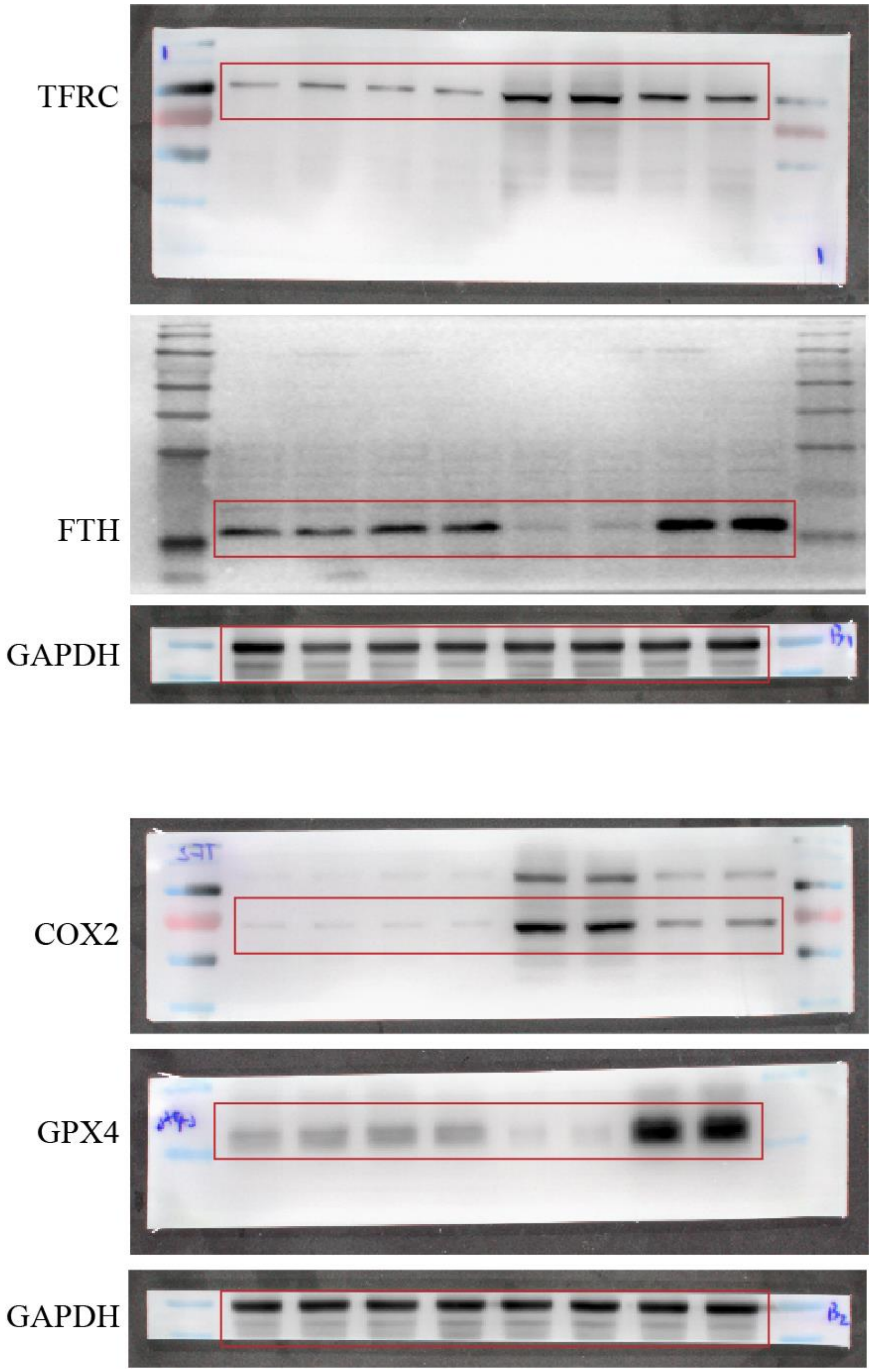

Figure 7

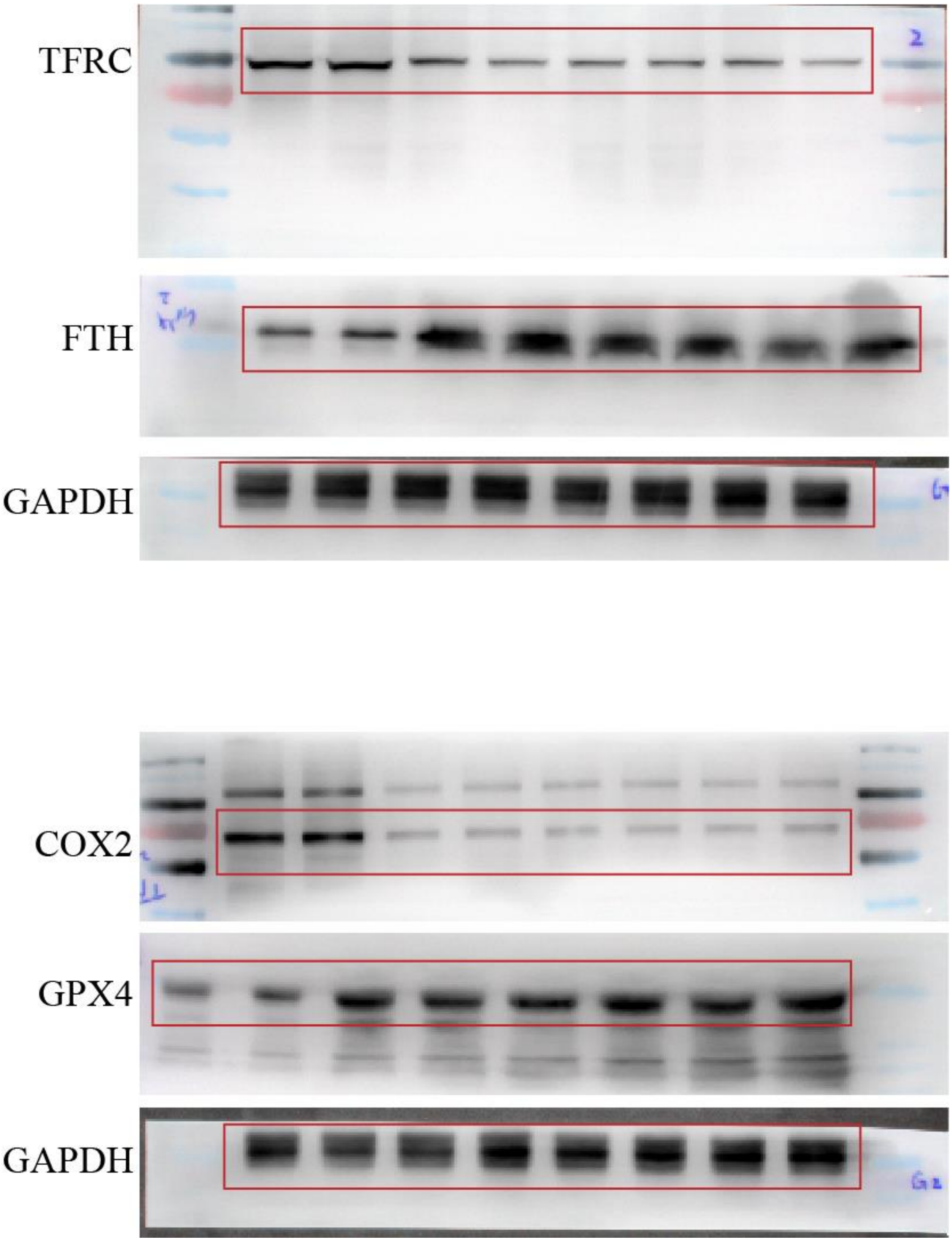

Supplement: Supplementary file 1 [file Data_Sheet_1.ZIP › Supplemental Figures/Fig S7 full gels.pdf]
