# Supplementary material for: Exosomes derived from pericardial adipose tissues attenuate cardiac remodeling following myocardial infarction by Adipsin-regulated iron homeostasis
Source: Front Cardiovasc Med. 2022 Sep 12;9:1003282. doi: 10.3389/fcvm.2022.1003282 (PMC9510661; doi:10.3389/fcvm.2022.1003282)
Supplement: Supplementary file 1 [file Data_Sheet_1.ZIP › Supplemental Figures/Fig S8 clinicaltrials.pdf]

Supplemental Figure 8.
The registration information of project in ClinicTrials (NCT04570527).

NIH U.S. National Library of Medicine

ClinicalTrials.gov

Find Studies | About Studies | Submit Studies | Resources | About Site

PRS Login

Optimized Cardioprotection Therapy in Obese Subjects With CAD

The safety and scientific validity of this study is the responsibility of the study sponsor and investigators. Listing a study does not mean it has been evaluated by the U.S. Federal Government. Know the risks and potential benefits of clinical studies and talk to your health care provider before participating. Read our disclaimer for details.

ClinicalTrials.gov Identifier: NCT04570527

Recruitment Status : Recruiting
First Posted : September 30, 2020
Last Update Posted : September 30, 2020

See **Contacts and Locations**

Sponsor:
Xijing Hospital

Information provided by (Responsible Party):
sunddong, Xijing Hospital

Study Details | **Tabular View** | **No Results Posted** | Disclaimer

How to Read a Study Record

Study Description Go to

Brief Summary:

The purpose of this study is to investigate the efficacy and mechanism of Adipokines Cardiac Protection in Obese Patients With coronary artery disease (CAD).

Condition or disease	Intervention/treatment
Coronary Artery Disease (CAD) (E.G., Angina, Myocardial Infarction, and Atherosclerotic Heart Disease (ASHD))<br>Obesity<br>Adiposis; Heart	Procedure: PCI

Detailed Description:

Adipokines (or adipocytokines) can be defined as a group of more than 600 bioactive molecules made from adipose tissue that acts as paracrine and endocrine hormones. Adipokines are involved in maintaining varieties of processes such as, appetite and satiety, energy expenditure activity, endothelial function, blood pressure, hemostasis, adipogenesis, insulin sensitivity, energy metabolism in insulin-sensitive tissues, fat distribution and insulin secretion in pancreatic β-cells. Adipokines may contribute to reduce scar formation and improve cardiac function in coronary artery disease (CAD). Reperfusion therapy such as percutaneous coronary intervention (PCI) should be administered to all eligible patients with CAD symptom. However, our previous work showed that obese patients may benefit more from PCI. Thus, the aim of the present study was to investigate the efficacy and mechanism of adipokines cardiac protection for obese and non-obese patients with coronary artery disease (CAD).

Study Design

Go to

Study Type :	Observational
Estimated Enrollment :	200 participants
Observational Model:	Case-Control
Time Perspective:	Retrospective
Official Title:	Effects and Mechanism of Adipokines Cardiac Protection in Obese Patients With Coronary Artery Disease (CAD)
Actual Study Start Date :	January 1, 2019
Estimated Primary Completion Date :	October 1, 2021
Estimated Study Completion Date :	December 30, 2022

Resource links provided by the National Library of Medicine NIH NLM

MedlinePlus related topics: Coronary Artery Disease

U.S. FDA Resources

Groups and Cohorts

Go to

Group/Cohort	Intervention/treatment

Obesity<br>BMI>=25	Procedure: PCI<br>percutaneous coronary intervention
Non-Obesity<br>BMI<25	Procedure: PCI<br>percutaneous coronary intervention

Outcome Measures

Go to

Primary Outcome Measures :

1. Number of patient death during the follow up period [Time Frame: 1 year]

Secondary Outcome Measures :

1. Left ventricular ejection fraction [Time Frame: 1 year]
2. Number of target vessel revascularization [Time Frame: 1 year]
3. Angina class according to the canadian cardiovascular society (CCS) classification [Time Frame: 1 year]
4. Scores on the Seattle angina questionnaire [Time Frame: 1 year]
5. six-min walk distance (6MWD) [Time Frame: 1 year]

Biospecimen Retention: Samples With DNA
Serum, Plasma

Eligibility Criteria

Go to

Information from the National Library of Medicine NIH NLM

Choosing to participate in a study is an important personal decision. Talk with your doctor and family members or friends about deciding to join a study. To learn more about this study, you or your doctor may contact the study research staff using the contacts provided below. For general information, Learn About Clinical Studies.

Ages Eligible for Study:	20 Years to 70 Years (Adult, Older Adult)
Sexes Eligible for Study:	All
Accepts Healthy Volunteers:	No
Sampling Method:	Non-Probability Sample

Study Population

Patients of CAD were enrolled in this study

Criteria

Inclusion Criteria:

coronary artery disease according to the WHO definition With or without percutaneous coronary intervention

Exclusion Criteria:

previous Coronary artery bypass grafting (CABG) cardiomyopathy atrial fibrillation or flutter previous heart surgery severe valvular heart disease disease of the hematopoietic system NYHA functional class IV heart failure at baseline severe renal, lung and liver disease cancer

Contacts and Locations

Go to

Information from the National Library of Medicine

To learn more about this study, you or your doctor may contact the study research staff using the contact information provided by the sponsor.

Please refer to this study by its ClinicalTrials.gov identifier (NCT number): ***NCT04570527***

Contacts

Contact: Dongdong Sun, MD, PhD 86 29 84775183 wintersun3@gmail.com

Contact: Wanrong Man, MD 86 29 84775183 Manwanrong@gmail.com

Locations

China, Shaanxi

Xijing hospital — **Recruiting**

Xi'an, Shaanxi, China, 710032
Contact: Dongdong Sun, M.D.,Ph.D 86 18691569930 wintersun3@gmail.com
Contact: Wanrong Man, M.D. 86 15352312311 manwanrong@gmail.com
Principal Investigator: Dongdong Sun, M.D.,Ph.D

Sponsors and Collaborators

Xijing Hospital

Investigators

Principal Investigator: Dongdong Sun, MD, PhD Xijing Hospital

More Information

Go to

Responsible Party: sunddong, Cardiology department, Xijing Hospital
ClinicalTrials.gov Identifier: NCT04570527 History of ChangesHistory of Changes
Other Study ID Numbers: OB-CAD
First Posted: September 30, 2020 Key Record Dates
Last Update Posted: September 30, 2020
Last Verified: September 2020

Studies a U.S. FDA-regulated Drug Product: No
Studies a U.S. FDA-regulated Device Product: No

Keywords provided by sunddong, Xijing Hospital:

- Coronary artery disease
- Adipokines
- Obesity
- Diabetes mellitus

Additional relevant MeSH terms:

- Coronary Artery Disease
- Myocardial Ischemia
- Coronary Disease
- Myocardial Infarction
- Heart Diseases
- Infarction
- Ischemia
- Pathologic Processes
- Necrosis
- Cardiovascular Diseases
- Arteriosclerosis
- Arterial Occlusive Diseases
- Vascular Diseases
