# Supplementary material for: Exosomes derived from pericardial adipose tissues attenuate cardiac remodeling following myocardial infarction by Adipsin-regulated iron homeostasis
Source: Front Cardiovasc Med. 2022 Sep 12;9:1003282. doi: 10.3389/fcvm.2022.1003282 (PMC9510661; doi:10.3389/fcvm.2022.1003282)
Supplement: Supplementary file 1 [file Data_Sheet_1.ZIP › Supplemental Figures/Supporting Information legend.docx]

**Supplemental Figure Legends**

**Supplemental Figure 1.**

**Representative immunohistochemistry images of adipose tissue obtained from** **wild type mice during the first 2 weeks of myocardial infarction (MI) and stained for Adipsin.**

**Supplemental Figure 2.**

**Isolation and Characterization of Exosomes.**

(A-C) Size distribution of the indicated exosomes as detected by Nanosight.

(D&E) The content of Adipsin in heart after injection of different exosomes.

Each group n=6. * *p*<0.05 vs Vehicle group, Ɨ *p*<0.05 vs SAT Exosomes group, § *p*<0.05 vs Epididymal-AT Exosomes group. Statistical analysis was performed by one-way ANOVA.

**Supplemental Figure 3.**

**Analysis of adipose-specific** **Adipsin overexpression mice.**

**Supplemental Figure 4.**

**Analysis of adipose-specific** **Adipsin knockout mice.**

**Supplemental Figure 5.**

Immunoprecipitation (IP) assay was carried out using Adipsin antibody or IgG (negative control antibody). Samples were electrophoresed and silver stained.

**Supplemental Figure 6.**

**The most effective short hairpin RNA (shRNA) recombinant adeno-associated viruses (AAV9) targeting Irp2 gene, and evaluate their interference efficiency.**

(A-B) Three pairs of siRNA sequences targeting mice Irp2 gene, then the most effective sequence (Irp2-1225) was designed, synthesized and then inserted into AAV9.

(C) The short hairpin RNA (shRNA) sequence was constructed.

(D) AAV9 information.

**Supplemental Figure 7.**

**Full images of Western blots.**

**Supplemental Figure 8.**

**The registration information of project in ClinicTrials (NCT04570527).**

**Supplemental Figure 9.**

**The validity of AVV9-IRP2 shRNA.**

(A-B) Western blots analysis the validity of AVV9-IRP2 shRNA in heart tissue 15 days after injection.

(C) Observation of heart tissue slides under fluorescence microscope 15 days after injection.

**Supplemental table 1.** **Baseline characteristics of participants.**

**Supplemental table 2. Mice survival data after myocardial infarction.**

**Supplemental table 3. RNA sequencing data.**

**Supplemental table 4. LC-MS/MS data.**

**Supplemental table 5. Real Time PCR Primers.**
