# Supplementary figures and images for: Exosomes derived from pericardial adipose tissues attenuate cardiac remodeling following myocardial infarction by Adipsin-regulated iron homeostasis
Source: Front Cardiovasc Med. 2022 Sep 12;9:1003282. doi: 10.3389/fcvm.2022.1003282 (PMC9510661; doi:10.3389/fcvm.2022.1003282)

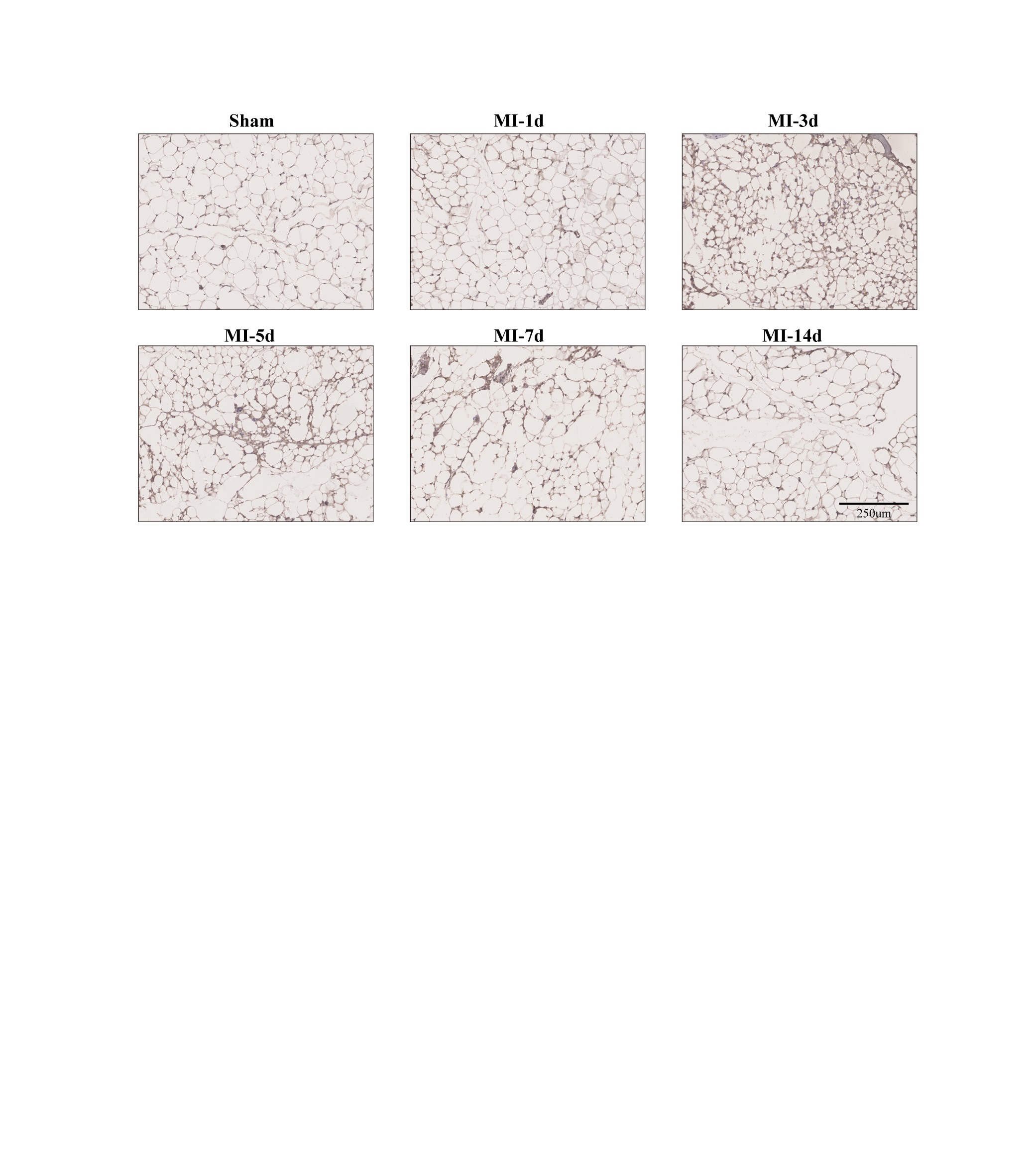

Supplement: Supplementary file 1 [file Data_Sheet_1.ZIP › Supplemental Figures/Fig S1 AT.tif]

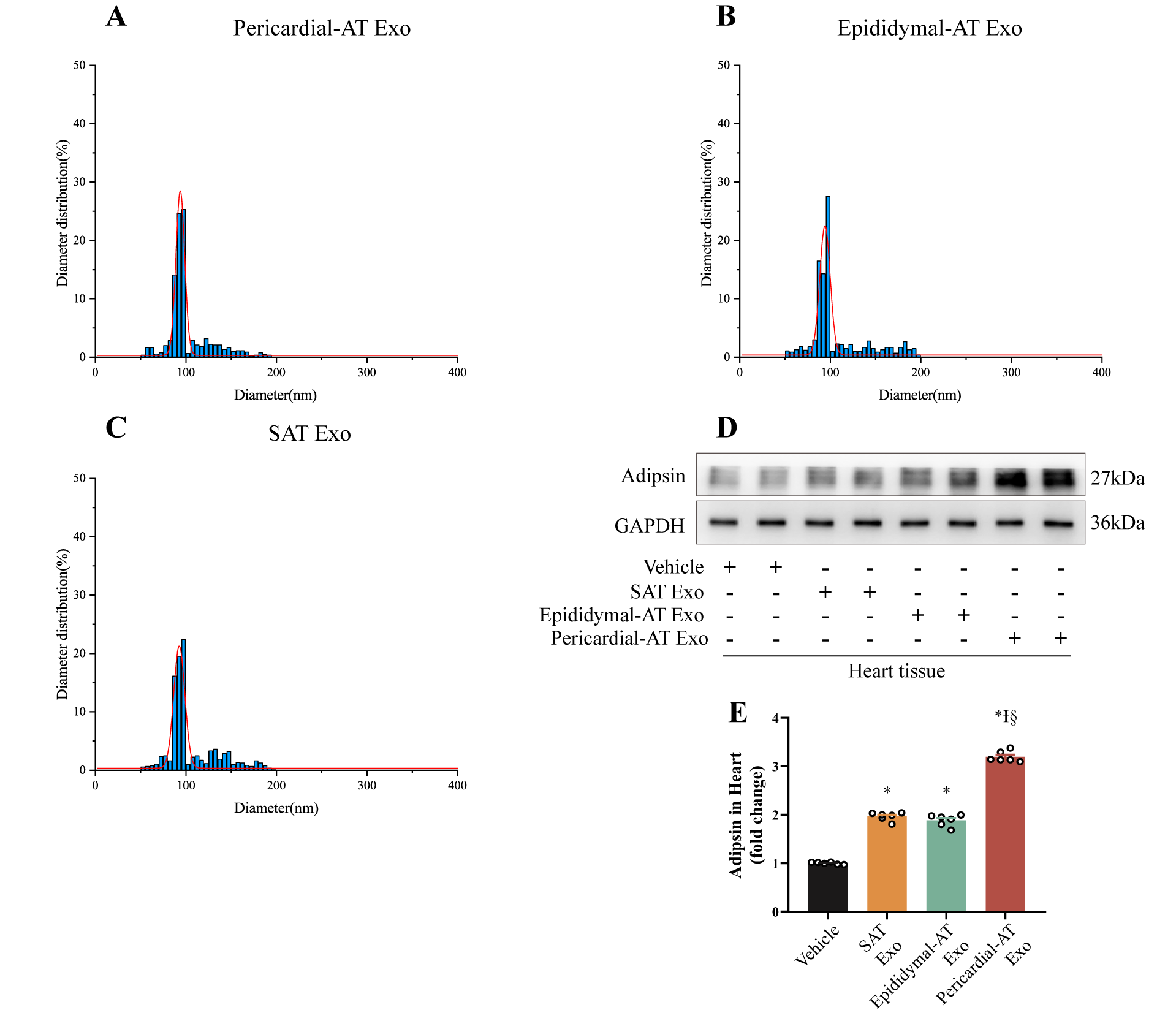

Supplement: Supplementary file 1 [file Data_Sheet_1.ZIP › Supplemental Figures/Fig S2 nano diameter.tif]

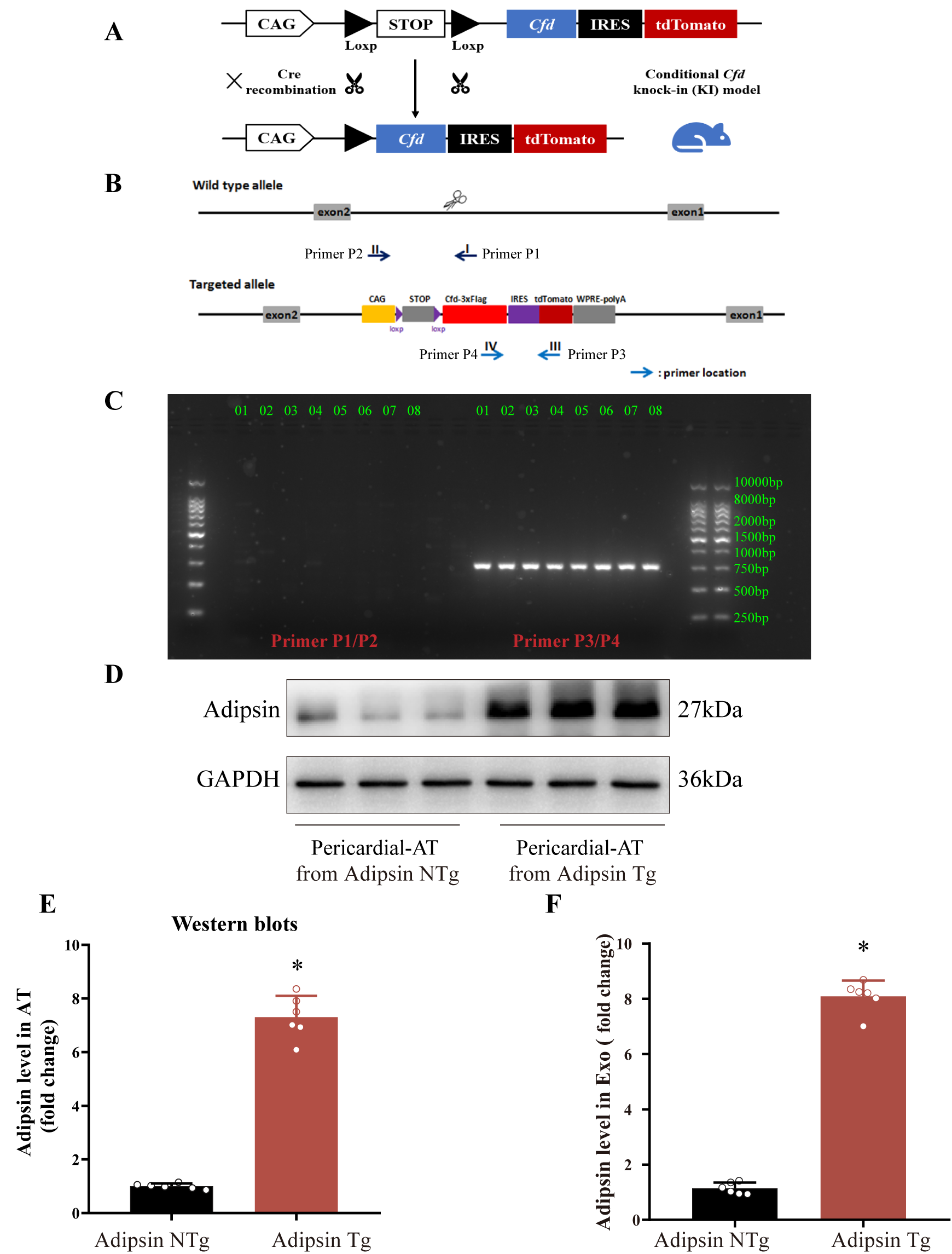

Supplement: Supplementary file 1 [file Data_Sheet_1.ZIP › Supplemental Figures/Fig S3 Tg.tif]

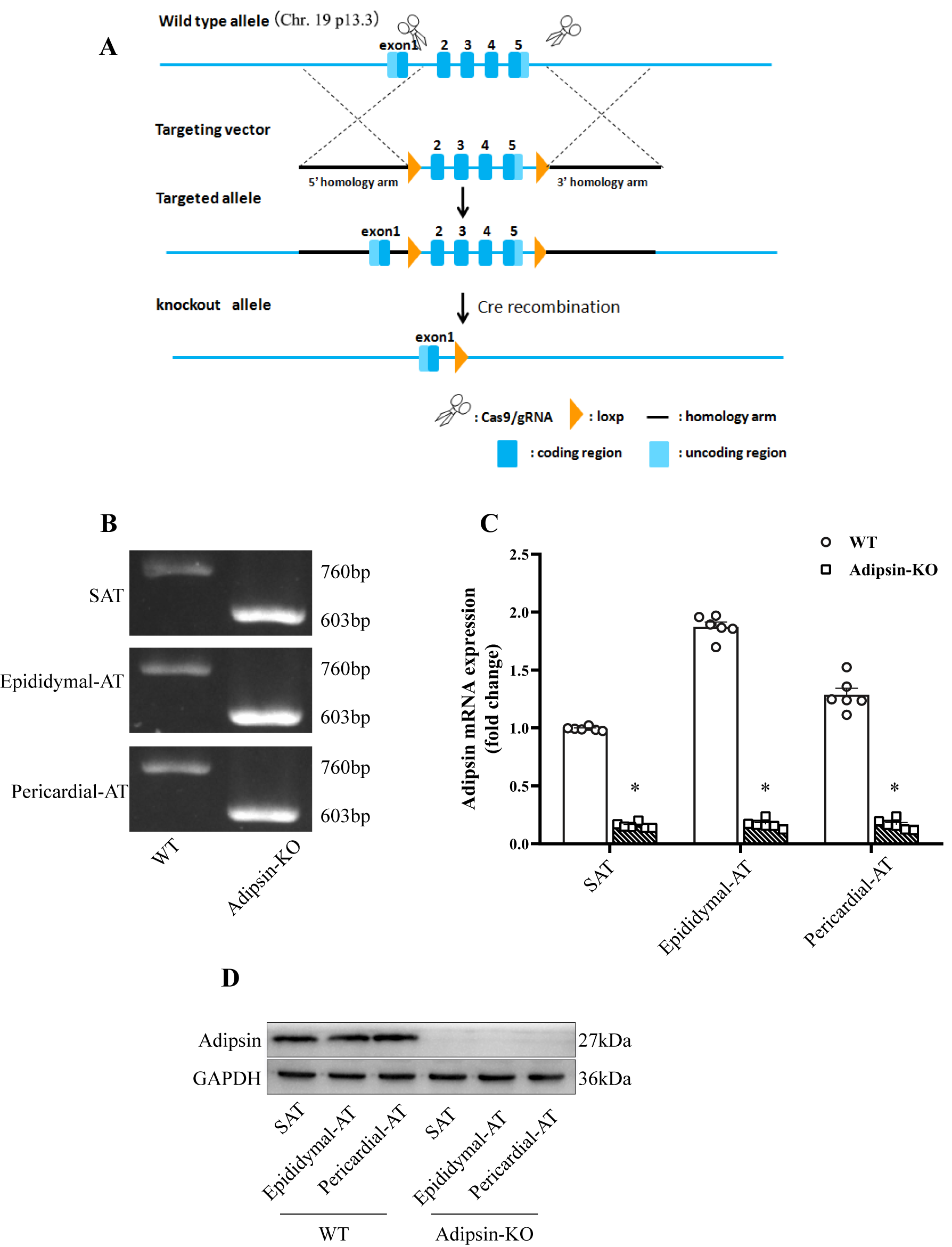

Supplement: Supplementary file 1 [file Data_Sheet_1.ZIP › Supplemental Figures/Fig S4 KO.tif]

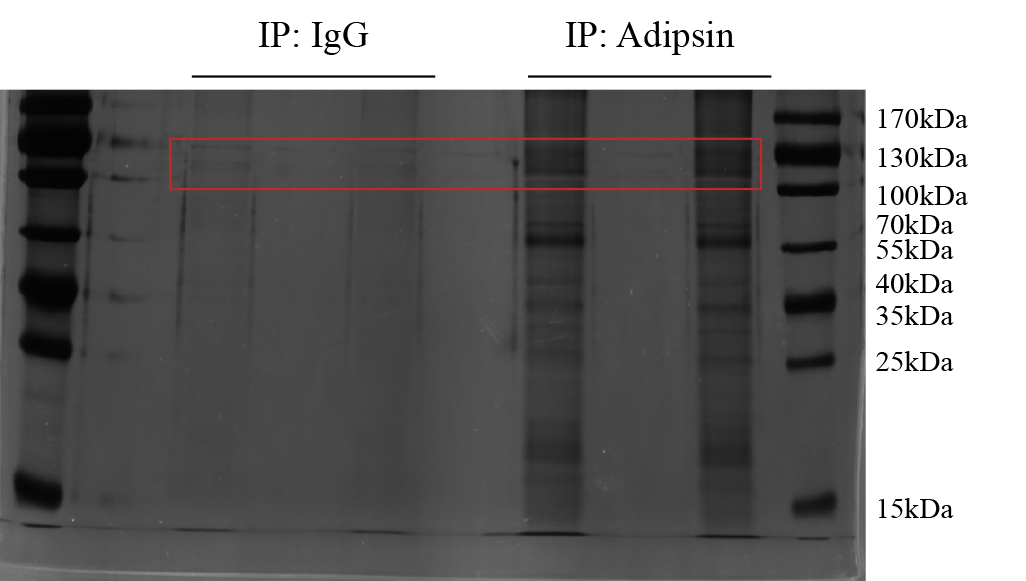

Supplement: Supplementary file 1 [file Data_Sheet_1.ZIP › Supplemental Figures/Fig S5 coipGel.tif]

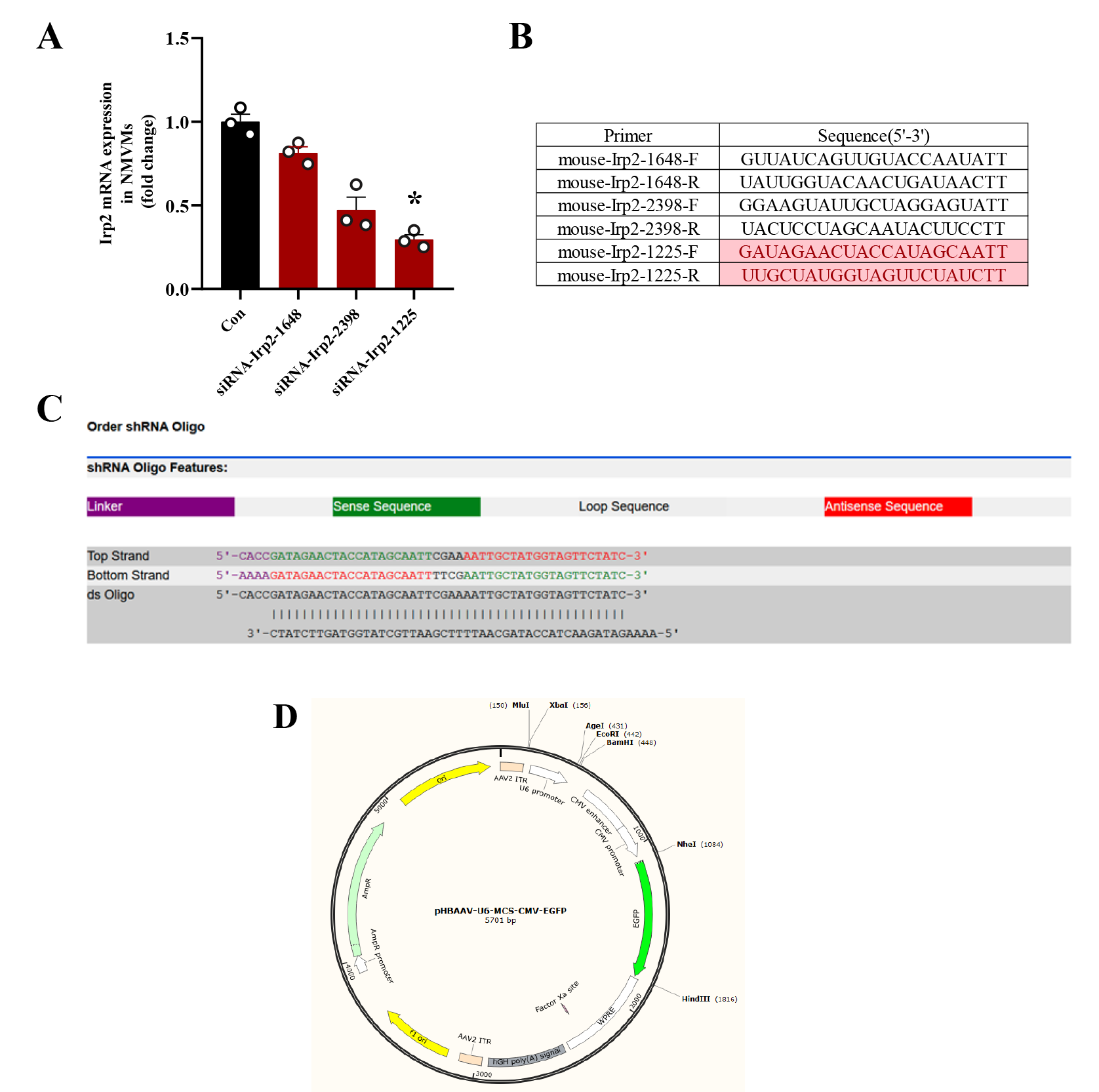

Supplement: Supplementary file 1 [file Data_Sheet_1.ZIP › Supplemental Figures/Fig S6 irp2 siRNA.tif]

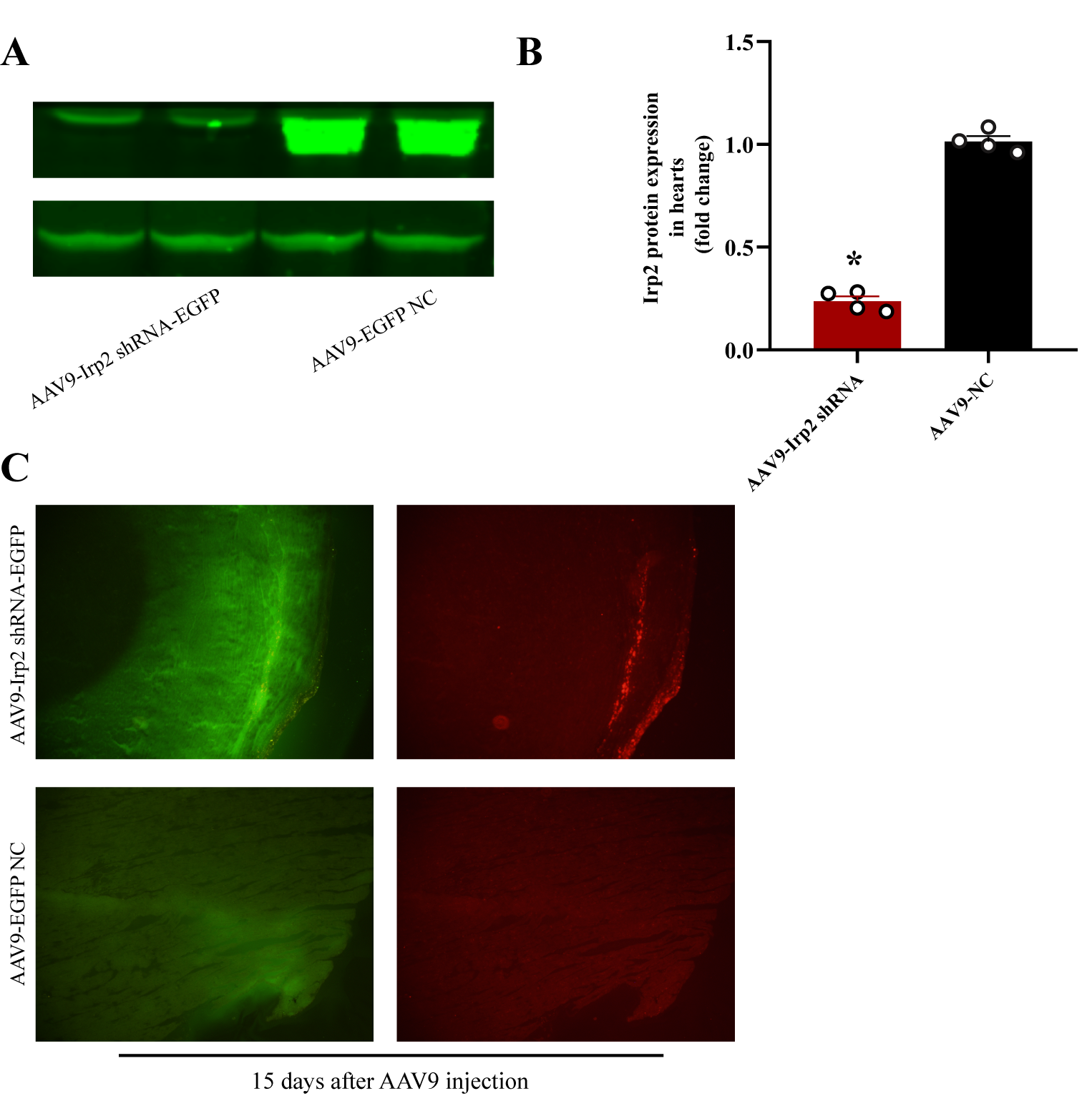

Supplement: Supplementary file 1 [file Data_Sheet_1.ZIP › Supplemental Figures/Fig S9 irp2 siRNA.tif]
